# Revealing the day-to-day regularity of urban congestion patterns with 3D speed maps

**DOI:** 10.1038/s41598-017-14237-8

**Published:** 2017-10-25

**Authors:** Clélia Lopez, Ludovic Leclercq, Panchamy Krishnakumari, Nicolas Chiabaut, Hans van Lint

**Affiliations:** 1 Univ. Lyon, IFSTTAR, ENTPE, LICIT, Lyon, F-69675 France; 2 Delft University of Technology, CITG, Delft, N-2600GA The Netherlands

## Abstract

In this paper, we investigate the day-to-day regularity of urban congestion patterns. We first partition link speed data every 10 min into 3D clusters that propose a parsimonious sketch of the congestion pulse. We then gather days with similar patterns and use consensus clustering methods to produce a unique global pattern that fits multiple days, uncovering the day-to-day regularity. We show that the network of Amsterdam over 35 days can be synthesized into only 4 consensual 3D speed maps with 9 clusters. This paves the way for a cutting-edge systematic method for travel time predictions in cities. By matching the current observation to historical consensual 3D speed maps, we design an efficient real-time method that successfully predicts 84% trips travel times with an error margin below 25%. The new concept of consensual 3D speed maps allows us to extract the essence out of large amounts of link speed observations and as a result reveals a global and previously mostly hidden picture of traffic dynamics at the whole city scale, which may be more regular and predictable than expected.

## Introduction

Studying human mobility in large cities is critical for multiple applications from transportation engineering to urban planning and economic forecasting. In recent years, the availability of new data sources, e.g. mobile-phone records and global-positioning-system data, has generated new empirically driven insights on this topic. A central question at large spatial and temporal scales is which (dynamic) components of human mobility can be considered as predictable and thus suitable for explanatory and predictively valid mathematical models, and which part is unpredictable. Earlier studies of human trips shows that traveled distance can be described by random walks and more precisely as Lévy-flights^1^. Latter studies partly amend this theory by recognizing some regularity features in peoples’ trips. Individuals obviously frequently move between specific locations, such as home or work^2^. Such patterns are also regular in time^3,4^ meaning that the most frequent locations are likely to be correlated with daily hours and dates. Regularity can also come from decomposition by transportation modes^5^. Human mobility can be studied at the microscopic level, i.e. through person trajectories, but also at the macroscopic level, for example by estimating commuting flows between different regions (origins to destinations) or on the different links of a transportation network^6,7^. Such collective mobility patterns can be explained for example by distances between regions^8,9^, trip purposes^10^ and road attractiveness related to road types, e.g. freeways, or locations, e.g. in major business districts^11^. Predicting commuting flows often requires local data for calibration^12^ meaning that results cannot easily be transferable to other regions or cities. Recent findings^13^, however, show that a scale-free approach corresponding to an extension of the radiation model can successfully be applied to commuting flow estimation. This means that some regular patterns can be observed also at the macroscopic level.

In this paper, we aim to pursue the investigation of regularity in macroscopic mobility patterns not by focusing on the commuting flow distributions; but on the resulting level of service of the transportation (road) network, i.e. on congestion patterns. Along with commuting flows, congestion patterns vary both within days and from day-to-day at large urban scales. It is common knowledge that some regularity happens as congestion is usually observed during peak hours on the most critical links of the network. In contrast to commuting flows, congestion patterns are more easily observed using real data as they only require speed information in the different network links. Nowadays, such information is easily accessible through different sensing technologies that are massively deployed in many cities. However, in large networks with speed data on hundreds (or thousands) of links over a large number of time periods, studying regularity and identifying distinct network congestion patterns is not an easy task to undertake: the challenge is to see the forest (regular large-scale traffic patterns) for the trees (many local pockets of queuing and congestion spillback processes). Here, we propose a new concept to address this challenge. We synthesize within days link speed data and simplify day-to-day comparisons, by means of so-called spatio-temporal speed cluster maps. Such 3D speed maps consist of a joined partition of space (road network links) and time (the different observations) into homogeneous clusters characterized by a constant mean speed. More precisely, such a partitioning should fulfill the following criteria: (i) all clusters should contain a single connected graph component meaning that all links are reachable within a cluster, (ii) the internal speed variance for all clusters should be minimized - the *intra-cluster homogeneity criterion* and (iii) the difference in speed between neighboring clusters should be maximized - the *inter-cluster dissimilarity criterion*.

Clustering is a common problem in different fields of engineering such as data mining^14^ or image segmentation^15^. Two recent and significant contributions in transportation for our work are (i) the application of the k-means algorithm^16^ to partition urban networks by considering spatial locations of the road as new features in the data and (ii) the definition of a similarity matrix between observations and the application of the Ncut algorithm^17^. These works result in 2D clusters, covering a spatial portion of transportation networks for a given time period. To obtain a picture of the traffic dynamics over different time periods, the algorithms are simply iterated for each time period without connecting the 2D clusters. Note that usual clustering works in transportation also include compactness as a requirement for clusters. This is because the main application is perimeter control. In this paper we present an algorithm that directly unravels traffic dynamics over both space and time. We favor connectivity - requirement (i) - rather than compactness for clusters, which makes more sense in 3D. To this end, we first determine which clustering method is the most efficient to cluster all time-dependent link speed observations into 3D speed maps, where we consider the intra-cluster homogeneity and inter-cluster dissimilarity criteria as well as the computational times to determine the optimal number of clusters. Second, we apply consensus learning techniques^18,19^ to summarize multiple 3D speed maps from a training set of days, into a single common pattern. Interestingly, such a meta-partitioning operation can be fulfilled with a very small number of groups. This means that the day-to-day regularity of daily congestion patterns can be easily revealed based on such a classification. Finally, we will show that using a single consensus pattern for each class of 3D congestion maps is sufficient to accurately estimate *in real-time* travel times in the city. This means that addressing congestion patterns directly at the whole city scale for all time intervals reveals a meaningful and accurate global picture of the city traffic dynamics that can be used as an efficient alternative to classical methods that process much more data at local and short-term scales.

## Results

**Figure 1:**
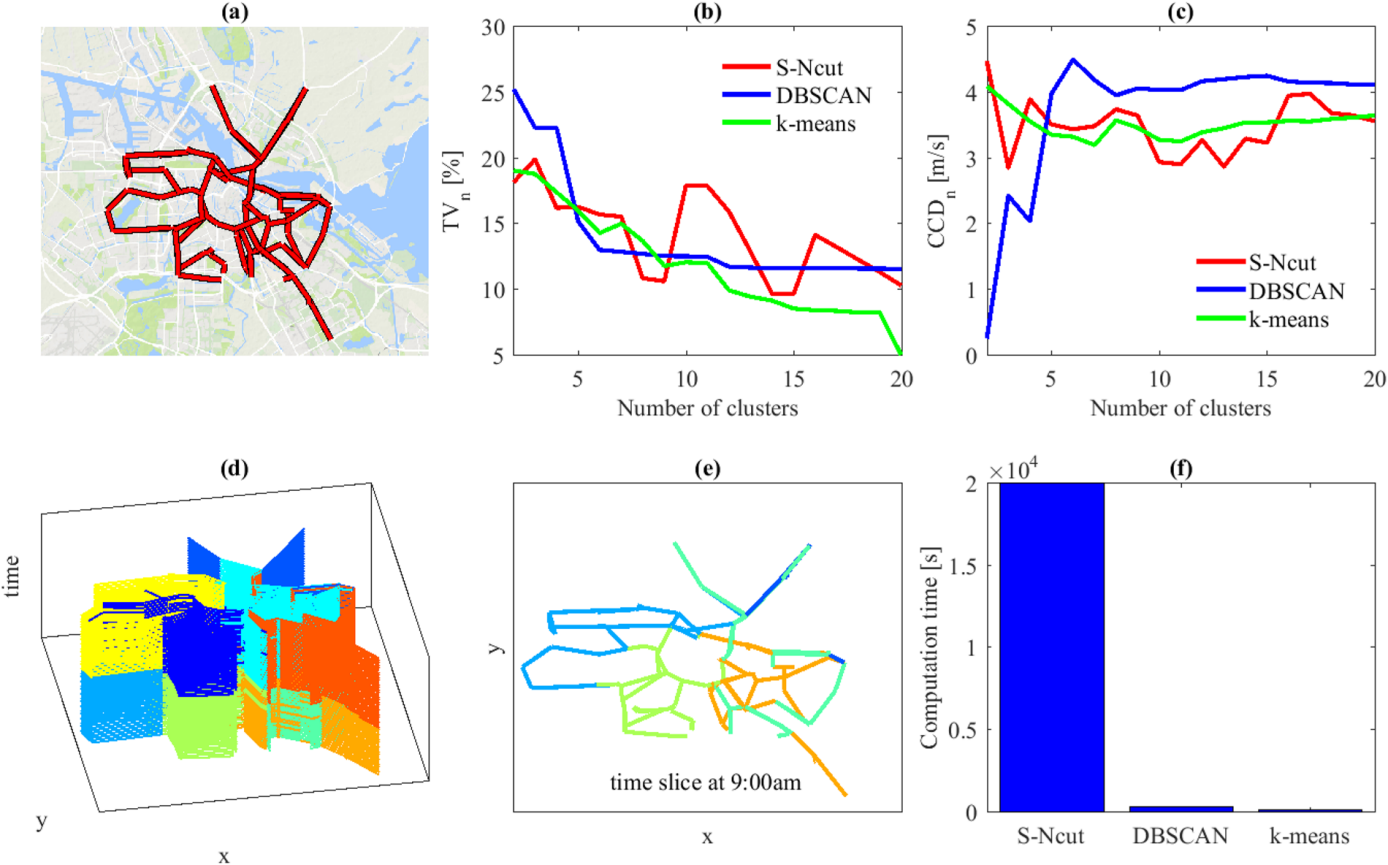
Link speed 3D clustering for one particular day. (**a**) Sketch of Investigated network - Amsterdam city (NL) - MapData @2017 Google (**b**,**c**) Evolution of the total variance (*TV*_*n*_) and the connected cluster dissimilarity (*CCD*_*n*_) with respect to the number of cluster for different clustering methods (**d**) Resulting 3D speed maps for 9 clusters (**e**) Slide of the 3D speed map for time period *t* = 9am (**f**) Computational times for different clustering methods and a targeted number of clusters equal to 9. Graphs (**b,c,f**) show that the clustering algorithms that do not consider the graph topology, i.e. the k-mean and the DBSCAN, blast the S-Ncut in terms of computational times with analogous *TV*_*n*_ and *CCD*_*n*_ results. DBSCAN appears very stable when the number of cluster exceeds 6. Selecting 9 clusters looks optimal for this dataset and network configuration.

Our case study corresponds to most of the major street network of Amsterdam city excluding the freeways, see Fig. 1(a). Whereas the original mapping of the inner city network contains over 7512 links, it is coarsened in this paper to 208 links and 214 nodes. Such an operation basically merges all successive links in the same direction between two intersections into a single one and disregards the internal links in the original mapping for intersections, see the method section. Mean speed information is available every 10 min between 7am and 3 pm for all 208 links during 35 days. This information is derived from license plate recognition systems at different critical points of the network. The methodology to derive link speed data from passing times, coarsen the network, and reconstruct missing data has already been published^20^. It should be noticed that all the methods elaborated in this paper can be applied to any set of time-dependent link speed data combined with the related connected graph (contiguous time intervals for the same network link should be connected by an edge) whatever the initial sensing method is.

### Clustering results for individual days

So, the initial data for a particular day is an undirected graph in which links are connected in space with their upstream and downstream neighbors following the road network, and in time by their immediate neighbors, i.e. the previous and the next time intervals for a given link. Each link is characterized by a spatial (*x*, *y*) position, a time and a speed value. Link directions are not considered during the clustering process because changes in traffic volume propagate forward while congestion propagates backward and we want to capture both phenomena. To obtain the 3D speed map related to such data, we first benchmark different clustering algorithms from the literature. We choose to oppose the most recent development in clustering for transportation networks, i.e. the Ncut algorithm with snake similarity also referred to as S-Ncut^17^ (see supplementary S1 for more details) with two simpler clustering algorithms, the k-means^21^ and DBSCAN^22^ algorithms, see the method section. The main difference between these, is that S-Ncut uses network topology when calculating the similarities between observations; whereas the two other methods simply use normalized Euclidean distances (regardless of topology) to balance both space, time and speed values. Note we weigh speed three times more heavily (*α* = 3) compared to space and time (vicinity) since our objective is to obtain clusters with a narrow speed distribution, see supplementary S2 for more rationales about the choice of *α*. The quality of the clustering results is assessed for a given number of clusters *n* through two indicators that relate to the *intra-cluster homogeneity* and the *inter-cluster dissimilarity* criteria respectively: the total within cluster variance (*TV*_*n*_) and the connected cluster dissimilarity (*CCD*_*n*_).

1 $$T{V}_{n}=\frac{1}{{\sum }_{i\mathrm{=1}}^{n}{n}_{i}}\frac{{\sum }_{i\mathrm{=1}}^{n}{n}_{i}{s}_{i}^{2}}{{s}^{2}};\quad CC{D}_{n}=\frac{{\sum }_{i\mathrm{=1}}^{n}{\sum }_{k\mathrm{=1}+i}^{n}{\delta }_{ik}\sqrt{{n}_{i}{n}_{k}}|{\bar{x}}_{i}-{\bar{x}}_{k}|}{{\sum }_{i\mathrm{=1}}^{n}{\sum }_{k\mathrm{=1}+i}^{n}{\delta }_{ik}\sqrt{{n}_{i}{n}_{k}}}$$

where *n*_*i*_ is the number of links in cluster *i*, ${\bar{x}}_{i}$ and *s*_*i*_ are respectively the mean and the standard deviation of link speeds for cluster *i*, ${\delta }_{ik}$ is equal to 1 only if clusters *i* and *k* have a common border and *s* is the standard deviation of link speeds for the whole network. Since we also impose that each cluster should contain a single connected graph component, clustering results should be post-processed, see supplementary S3. Note that S-Ncut results, even though the method includes topological considerations to calculate similarity between observations, also require post-processing, see supplementary S1. Post-processing has very little impacts on *TV*_*n*_ and *CCD*_*n*_ values for S-Ncut. It deteriorates *TV*_*n*_ values and to a lesser extent also *CCD*_*n*_ values for DBSCAN and k-means methods, see supplementary S3. This is not surprising as these two methods only account for proximity (distance between links) and not for connectivity within a cluster. In the end, what is important to assess the quality of a method is to compare *TV*_*n*_ and *CCD*_*n*_ values after post-processing when we are sure that *connectivity* - requirement (i) - is verified.

Clustering results after post-processing are presented for a randomly selected day among the 35 available in Fig. 1(b–f). The evolutions of *TV*_*n*_ in Fig. 1(b) and *CCD*_*n*_ in Fig. 1(c) are comparable for all three methods, although k-means can be identified as the best method to minimize *TV*_*n*_, and DBSCAN appears slightly more efficient in maximizing *CCD*_*n*_. DBSCAN also appears to provide more stable (i.e. monotonically decreasing) results for increasing cluster numbers than the other two. However, the *TV*_*n*_ and *CCD*_*n*_ values are not sufficiently different to provide conclusive evidence that one method is better than the other two. What can be concluded is that the S-Ncut algorithm has much higher computational times than the other two, which disqualifies the method since clustering has to be repeated for multiple different days. Both k-means and DBCAN are over 20 times faster than S-Ncut on the same computer, see Fig. 1(f). Finally, Fig. 1(b,c) highlight that improvements to *TV*_*n*_ and *CCD*_*n*_ values tend to significantly reduce when the number of cluster exceeds 9 to 10. This means that for this particular day, the optimal number of clusters can be fixed to 9. The resulting 3D speed map is presented in Fig. 1(d). A 3D video is also visible on the data repository website, see additional information. In Fig. 1(e) a slice at time *t* = 9am is shown to illustrate the clustering results in detail. Note that links from the same cluster may look not connected because of the slicing but they are of course connected through time links and different time periods.

**Figure 2:**
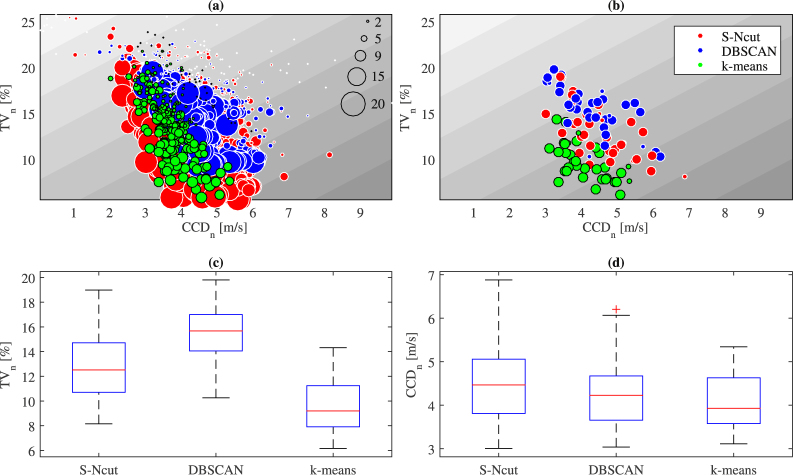
Clustering results for all 35 days. (**a**) Clustering efficiency with respect to the number of clusters (**b**) Clustering efficiency for a number of clusters equal to 9 (**c,d**) *TV*_*n*_ and *CCD*_*n*_ values (respectively) for all days and 9 clusters. (**a**) Shows that S-Ncut provides the best results compared to the other two methods when the number of cluster is large (above 15). However, when the number of clusters is reduced to 9 (**b**), k-means provides in general the lowest *TV*_*n*_ values while leading to similar *CCD*_*n*_ values than S-Ncut and DBSCAN. This is confirmed by (**c**) and (**d**) that show *TV*_*n*_ and *CCD*_*n*_ distribution for all methods when the number of cluster is 9. More interestingly, by comparing (**b**) and (**a**), it appears that k-means with only 9 clusters usually lead to close results compared to S-Ncut with a significant higher number of clusters. So, we define as 9 the optimal number of clusters for all days.

Figure 2 now presents the clustering results for all 35 days. Figure 2(a) shows that S-Ncut and k-means generally outperform the DBSCAN method with lower *TV*_*n*_ values. The score on *CCD*_*n*_ values is much less decisive. However, when reducing the number of clusters to 9, and testing all methods with this same number of clusters, k-means clearly outperforms the other methods over all 35 days. Interestingly, when comparing Fig. 2(b) to Fig. 2(a), one can observe that for this relatively low number of clusters (9), using k-means results in *TV*_*n*_ values that are very close to the best results obtained with any of the other two methods for larger number of clusters. Figure 2(c) and (d) provide a direct comparison of the three methods with respect to minimizing *TV*_*n*_ and maximizing *CCD*_*n*_ for *n* = 9. The k-means method generates a distribution of *TV*_*n*_ values for all days that is significantly better (lower) than both other methods. The distribution of *CCD*_*n*_ with k-means is not the best (the highest), but it is very close to what is obtained with the best methods for this indicator, i.e. the S-Ncut see Fig. 2(d). Since k-means is the most economical method in terms of computational cost, we can conclude that it must be favored to obtain 3D speed maps in this case. Furthermore, the results provide evidence to fix the optimal number of 3D clusters to 9 for our case study.

### Classification of multiple days to identify consensual congestion patterns

**Figure 3:**
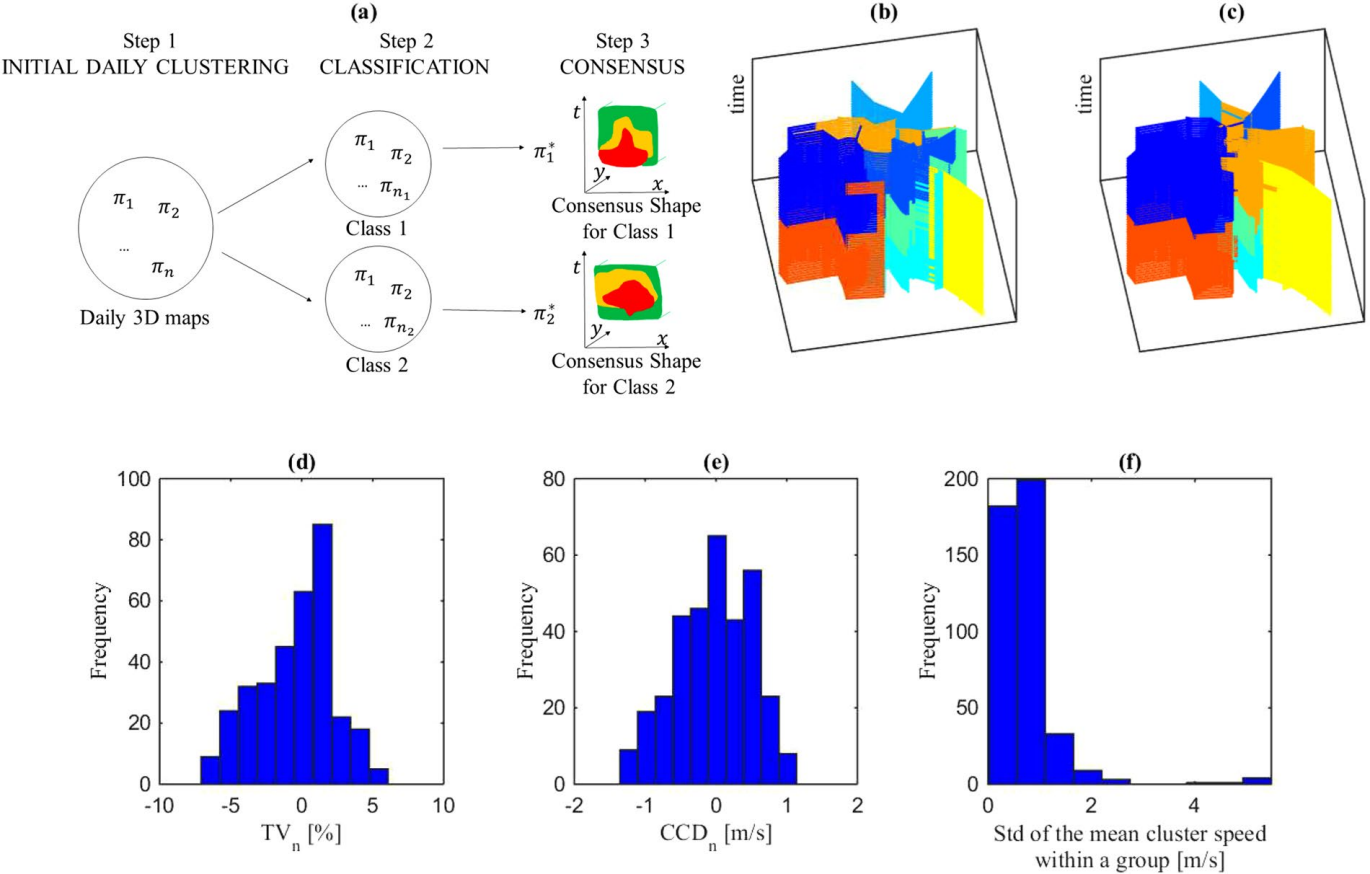
Classification of multiple days and congestion patterns identification for training sets. (**a**) The three steps to obtain consensual 3D speed maps (**b**) Original clustering for a particular day (**c**) Consensus clustering for the same day (**d**) Variation of *TV*_*n*_ between the original and the consensual cluster shapes for all days and all replications of the training set (**e**) Variation of *CCD*_*n*_ between the original and the consensual cluster shapes for all days and all replications of the training set (**f**) Distribution of the standard deviation of the mean cluster speed within a group of days (one value per cluster ID, group and replication). (**d**) and (**e**) Show that in most case switching from the original to the consensus shapes for a day has minor to acceptable impacts on the *TV*_*n*_ and *CCD*_*n*_ values. This means that the consensus shapes can be considered as a good proxy for the clustering of each day. (**f**) Shows that the consensus shape is also relevant to identify homogeneous regions in speed within a group as the standard deviation of the mean cluster speed remains below 0.5 m/s for the vast majority of cases.

Now our objective is to find commonalities in the 35 daily congestion patterns, and, ideally, summarize these with a fewer number of “consensual” patterns. To this end, we first have to define a common link network for all the 35 days, see supplementary S4. This is necessary because some links may have insufficient observations on particular days to be assigned with a significant value. The procedure has 3 main steps as outlined in Fig. 3(a). In step 1 we obtain 3D speed maps related to each daily pattern, by running the k-means algorithm with 9 targeted clusters over all the 35 days of the dataset. After this, each observation, i.e. a couple composed by a link and a time period, is assigned a cluster ID *i*. Each day *k* can then be synthesized into a single ordered vector of all observations *π*_*k*_, whose values are the cluster ID. To compare two different days *π*_*k*_ and *π*_*l*_ and assess if their 3D speed maps have similar shapes, we use the normalized mutual information (NMI) indicator. It has been designed to assess the proximity between two clustering results^18,23^.

2 $$NMI({\pi }_{k},{\pi }_{l})=\frac{I({\pi }_{k},{\pi }_{l})}{\sqrt{H({\pi }_{k})H({\pi }_{l})}}=\frac{H({\pi }_{k})+H({\pi }_{l})-H({\pi }_{k},{\pi }_{l})}{\sqrt{H({\pi }_{k})H({\pi }_{l})}}$$

where *I*(*π*_*k*_, *π*_*l*_) is the mutual information between *π*_*k*_ and *π*_*l*_, which measures the mutual dependence between two random variables^18^, $H({\pi }_{k})$ is the entropy of *π*_*k*_ and $H({\pi }_{k},{\pi }_{l})$ is the joint entropy of *π*_*k*_ and *π*_*l*_. Calculating the NMI for all day couples allows us to define a similarity matrix. We can then classify the whole set of days using the Ncut algorithm^15^, see step 2 in Fig. 3(a). More specifically, we apply a classical cross-validation approach by randomly splitting our 35 days into a training set of 28 days and a validation set of 7 days and considering 12 replications in total. The purpose of the validation set will be explained later. We test a partition of the 28 training days into 2 and 4 groups for all replications of the training set. It appears in all cases that 4 groups lead to better results, see supplementary S5. All four groups appear homogeneous with high mean NMI values inside a same group (usually higher than 0.6) and low differences between the maximum and the minimum NMI values (usually below 0.24). When looking at the day labels (Monday, …) within the four groups, no clear pattern appears. The major conclusion at this stage is that the 28 days can be classified into only 4 groups, which exhibits close 3D speed map shapes. We are now going to adjust the cluster shapes of the days belonging to the same group to obtain a unique consensual shape that can be applied within the group.

The consensus clustering problem consists in identifying the most representative partition from a group of partitions^18,24^. The best of K (BOK) algorithm^19^ can be used to determine the median partition *m* (the 3D speed map shape of a single day in our case) that maximizes the total similarity *TS* with all the other days belonging to the same group:

3 $$TS=\sum _{k\mathrm{=1}}^{a}NMI({\pi }_{m},{\pi }_{k})$$

where *a* is the number of days in the targeted group, ${\pi }_{m}$ is the vector resulting from the initial clustering (3D speed map) for the median partition, and ${\pi }_{k}$ the same vector but for each other day of the same group. The median partition can be further improved (increasing *TS*) by moving some of its elements from one cluster to another, i.e. changing the cluster ID of some elements in the vector. To realize such an optimization, we apply the one element move (OEM) algorithm^19^. It consists in randomly changing the label of one element of the vector and assess if such a change improves the TS value. The algorithm stops when TS has not been improved for a while. Determining the consensus shaping for all 4 groups corresponds to the final step 3 of the data processing, see Fig. 3(a). Figure 3(b) and (c) illustrate the difference between the original cluster shape of a particular day and the consensual shape resulting from the processing of all days in the same group. Figure 3(d) and (e) respectively show the variations of the *TV*_*n*_ and *CCD*_*n*_ values when comparing the consensus cluster shape with the original one for all the training days and all replications. It appears that the *TV*_*n*_ values significantly deteriorate (increase by more than 2%) for only 15% of the days while the *CCD*_*n*_ are significantly worse (decrease by more than −0.5 m/s) for only 20.8% of the days. Even for the days that see a significant change in the clustering quality, the final values related to the consensual shape remain always acceptable. This means that the consensual shape is relevant to describe in a unique and common manner the congestion patterns of the same group of days. Since classification into 4 groups appears sufficient, the conclusion is that the 3D speed maps of the 28 training days can be synthesized into only 4 different consensual congestion patterns. For now, the groups and the consensual shapes have been defined based on the initial cluster shapes without using the link speed information. The remaining question is to assess whether the consensual shapes are also relevant to define homogeneous regions in speed for each group of days. Because the consensual shape is the same within a group, it is easy to calculate the mean speed for each of the 9 cluster IDs and each day. Figure 3(f) shows the distribution of the standard deviation of such a mean cluster speed among all days belonging to the same group. Such a calculation has been performed for all replications of the training set. It turns out that 37% of the standard deviation values are below 0.5 m/s and the vast majority (85%) is below 1 m/s. This means that the mean cluster speeds are very close for the same cluster ID among the days of the same group.

This is a major result because it implies that the consensual shape is also relevant to summarize the speed profile observed in the network over time for a same group of days. For a given group, we can associate to each consensual cluster ID the mean of the mean cluster speeds for each day and so, obtain a single 3D speed map that defines the congestion pattern of this group. In other words, all days of the same group can be synthesized into no more than 9 cluster shapes and 9 mean speed values. For our case study (the Amsterdam network), 4 consensual 3D speed maps look sufficient to capture the functioning of the entire work network over the 35 days and to get a full overview of the dynamic traffic conditions within the major road network of the city. This is strong evidence for a high degree of regularity and predictability of macroscopic traffic conditions in this network.

### Application to real-time travel time prediction

We are now going to take advantage of the above major result to propose a fresh new look on a classical and popular problem in transportation systems, i.e. travel time prediction. This problem has been extensively investigated in the transportation literature using both (simulation) model-based and data-driven approaches as shown by recent review papers^25,26^. Model-based approaches use network traffic flow models in conjunction with data assimilation techniques such as recursive Bayesian estimators to predict the traffic state and the resulting travel times in networks^27–29^. Data-driven approaches use general purpose parameterized mathematical models such as (generalized) linear regression^30,31^; kriging^32^; support vector regression^33^; random forest^34^; Bayesian networks^35^; artificial neural networks, e.g. dynamic^36,37^ and (increasingly often) deep learning architectures^38,39^; and many other techniques to capture (learn) from data the correlations between traffic variables (speed, travel time) over space and time. When reviewing the literature, there are many more approaches reported for estimation and prediction on freeway corridors, than for mixed or urban networks, which we hypothesize is due to two reasons. First, until recently, insufficient data sources were available for such large-scale urban prediction models. Additionally, and more tentatively, the urban prediction problem is a more complex problem to address than the freeway prediction problem because there are many more degrees of freedom that govern the underlying local traffic dynamics (e.g. intersection control, crossing flows, high-frequency queuing also under free flowing conditions, much more route alternatives, etc), and thereby also the dynamics of speed and travel time. Recently, both model-based^29,40^ and more unified and systemic data-driven approaches^38,41–43^ have been proposed that, at least in principle, can be used to predict traffic variables in large-scale urban networks. However, when applied to large-scale networks, both model-based and data-driven approaches are indeed computationally complex, and methodologically cumbersome due to the high number inputs and parameters that continuously need to be calibrated and validated from data.

**Figure 4:**
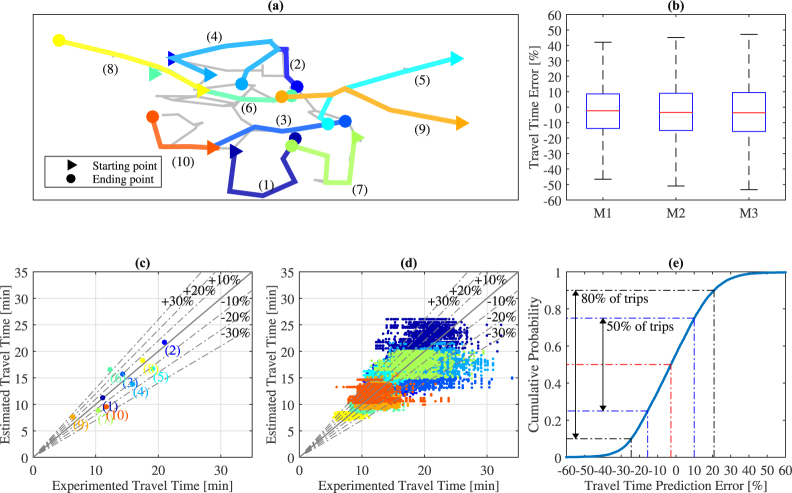
Travel time estimation based on congestion patterns. (**a**) Map of the probe trips (**b**) Travel time estimation errors for all probe trips and all training days considering the three estimation methods: M1, link speed is the mean speed in the original cluster; M2, link speed is the mean speed in the consensus cluster; M3, same as M2 but the mean speed is calculated over all days of the same group (**c**) Estimated vs. experimented travel times for the 10 probe trips, one validation day and a departure time equal to 9am (**d**) Estimated vs. experimented travel times for the 10 probe trips, all validation days and all departure times (**e**) Distribution of the travel time estimation errors for the 10 probe trips, all validation days and all departure times. (**b**) Shows that travel time errors are in most case relatively low. Averaging speed within each cluster has the highest contribution to errors. Interestingly, using the consensus cluster shape ($M1\to M2$) and the average of all days within a group ($M2\to M3$) have very impacts on errors. (**c**–**e**) Show that travel time predictions based on assigning a new day to an historical group and using the consensus cluster shape and the mean cluster speed of the group are very good for most probe trips.

As an alternative, we propose a very simple and systemic approach that uses the consensual congestion patterns obtained in the previous section. First, let us define a number of probe trips that we will use for investigating the methods and the validation. Based on the network map, we define 10 trips that cover most of the network links, see Fig. 4(a). A virtual probe vehicle is launched every 10 min over the time period between 8 am and 2 pm and its travel time is calculated based on the time-dependent link speed information of the studied day. This defines for each day 370 probe trips characterized by the travel time that a vehicle would have experimented for this trip and this departure time. Note that travel time calculations are made on the directed version of the road network graph while the initial and consensual clustering were made without considering directions. First, we are going to investigate if the mean speed values related to the 3D congestion maps can be considered as a good proxy for the travel time calculation. For now, only the days included in the 12 different training sets are considered because their group label and thus their consensus clustering shapes are known. We define three methods to estimate the travel time depending all the options we have to define congestion maps:

- M1: initial cluster shape of the day + link speeds equal to the mean speed value of all links in each initial cluster and the same day
- M2: consensus cluster shape of the group + link speeds equal to the mean speed value of all links in each consensus cluster and the same day
- M3: consensus cluster shape of the group + link speeds equal to the mean speed value for all links in each consensus cluster over all days of the group.

Figure 4(b) shows the distribution (box plot) of the travel time estimation errors for all probe trips, all training days and the three methods. It appears that averaging the link speeds within each initial cluster (M1) obviously introduces errors in the travel time estimation: (i) the mean and median errors are respectively equal to −2.0% and −2.3%, and are thus close to 0 (ii) 50% of the probe trips (25th to 75th percentiles) have errors between −13.7% and 8.6% and (iii) 80% of the probe trips (10th to 90th percentiles) have errors between −22.1% and 17.6%. Interestingly, most of the errors come from the averaging process within the cluster: when switching to the consensus cluster shape (M2) or replacing mean cluster speeds of the day by the mean cluster speeds of the group of days (M3) leads to error distributions that are very close to what is observed for (M1). In particular, for M3, the mean and median error values are respectively −2.7% and −3.6%, 50% of the probe trips exhibit errors between −15.7% and 9.4% and 80% of the probe trips have errors between −23.9% and 19.2%. These results are fundamental because they first confirm from another perspective (here the travel time estimation) that consensus congestion maps with mean speed in each cluster determined over a similar group of days are very relevant to synthesize the traffic congestion pulse at the city level. We see no discrepancy when switching from M2 to M3 meaning that all days of a group have similar speed behavior within each consensus cluster. As no discrepancy is observed when switching from M1 to M2, the consensus shape appears to be a good proxy to partition all the days of the same group. Together, these two results demonstrate that the consensus cluster decompositions are relevant not only in terms of shape but also in terms of mean cluster speed values and provide a unique and systemic picture of what happen for all days belonging to a same group. Note that the same graph as Fig. 4(b) but with absolute travel time errors is presented in supplementary S6.

The previous analysis provides the rationale for a simple, systemic and real-time travel time prediction method for new days belonging to the validation sets. For a new day, M1 and M2 are no longer relevant because they require the data of this particular day. However, M3 still holds as long as the new day can be assigned to an existing group obtained through historical analysis, i.e. over the training set. The only missing component is a method to allocate in real-time the current observations of a new day to an existing group. Knowing the group, the predetermined consensus cluster shape and the related mean speed values for each cluster can be applied to predict the future travel times. Here, we propose a simple method with very low computational times to match a new day with an existing group. This method only requires the link speed information until the actual time *t* of the new day. First, we reduce the consensual cluster shape of each historical group (4 in our case) to the period of time between 7am and *t*. Then, we apply all restricted consensual cluster shapes both on the new day data and on the consensus map of the related group. Mean speed values for the same cluster *i* in the new day ${x}_{i,g}$ and the consensus ${y}_{i,g}$ are compared. The optimal group index g* minimizes the Euclidean speed distance between the current day and the group:

4 $${g}^{\ast }=\mathop{{\rm{\arg }}\,{\rm{\min }}}\limits_{g}(\frac{1}{n}\sum _{i\mathrm{=1}}^{n}{({x}_{i,g}-{y}_{i,g})}^{2})$$

Note that the number of clusters *n* within the restricted time period (7am-*t*) can be lower than 9 in particular at the beginning of the day where all 3D patterns have not yet necessarily appeared. In practice, we can refresh the assignment of the new day to a group every hour starting at 8am, and assess the travel time predictions on the probe trips where a new virtual vehicle starts every 10 min. Figure 4(c) shows the results for a particular validation day and all trips starting at 9 am. It appears that, even if the reference time period to assign the day to a group is short (here 7 am–9 am), the predicted travel times are close to the experimented one for all trips, i.e. all error values but one fall between −20% and 20%. Note that the travel times are simply calculated using the link speed values of the full day since we are not testing the application in real-time here. This means that we already know all the link speed information for the validation days on the contrary to a real-time implementation where the future is unknown. Figure 4(d) shows exactly the same results but now for all validation days (7 days and 12 replications meaning 84 in total) and all departure times between 8am and 2 pm. Again, a large fraction of the travel time predictions (72.1% of the total probe trips) exhibit errors between −20% and 20% and almost all (91.9%) fall within an ±30% error margin. Despite its simplicity and its very low computational cost, the proposed method leads to accurate travel time predictions for most trips. This is confirmed by Fig. 4(e), which shows the cumulative distribution of all prediction errors. The mean and median values are equal to −2.2% and −2.7%, 50% of the probe trips experiments errors between −15.5% and 10.0% and 80% of the probe trips have errors between −24.5% and 20.8%. The counterpart of Fig. 4(e) with absolute travel time errors is provided in supplementary S6.

## Discussion

In this paper, we questioned the regularity of day-to-day mobility patterns at the macroscopic level. The global analysis of Amsterdam link speed data over 35 days shows a high degree of regularity when comparing the daily congestion patterns. In our case, four consensual 3D speed maps related to four groups of days are sufficient to describe the daily traffic dynamics at the city scale. This is remarkable given the fact that these consensual 3D speed maps are very parsimonious: for our case study, they consists of 9 clusters (collections of link and time ID) only, each characterized by a single mean speed value. A key contribution here was to use consensus learning methods to turn the cluster shapes of different days belonging to the same group into a single common pattern. Note that if more days are available for the learning, it is possible to keep the same level of quality for the consensual shape by increasing the number of groups. The NMI index permits to monitor the level of dissimilarity within a group of days and determine if a group should be split or not. This paper has thus demonstrated that consensual 3D speed maps are a new and very powerful tool to capture the congestion pulse in one shot at the whole city scale. It should be noticed that some factors that have not been observed during our sample of 35 days may influence the regularity of congestion patterns. From our experience, we can mention adverse weather conditions; exceptional (large cultural) events; or incidents as sources of major disruptions in the network. Over longer time periods, during which such situations are observed multiple times, the number of groups will increase to accommodate the resulting broader array of patterns, and most likely some regularity patterns with low frequency of appearance will emerge. Only the consequences of very rare or specific events are fully unpredictable.

A second major finding in this paper is that these consensual 3D speed maps allow us to design a simple and systemic method to predict travel times in an entire city. In this method first prevailing link speed observations are matched to an existing group of days. Subsequently, the consensual 3D speed map related to this group is used to predict the travel time of any trip within the city. This method is *real-time and practice ready* as the matching step is computationally lightweight. It corresponds to the selection of the best consensual 3D speed maps among the existing group of days based on the comparison of the mean speed in each cluster. In our data set, we succeeded in making travel time predictions for more than 84% of the trips with an absolute error lower than 25%, which is sufficient for most potential practical applications like traffic information provision, route guidance, traffic control and management, or optimizing good deliveries and solving vehicle routing problems.

The methodology presented in this paper to derive consensual 3D speed maps can be easily implemented in the real field. Link speed data at a granularity of say 1–10 minutes become more and more readily available thanks to advances in estimation methods using classical data (induction loops, cameras) and new data sources based on crowd-sourcing (mobile-phone records, GPS tracking). One clear direction for (methodological) improvement relates to decreasing computational costs, particularly when determining the initial 3D speed map for a new day on much larger networks in terms of number of links. Our aim was to make the case for 3D patterns as a new way to identify large-scale regularity in traffic networks and it turned out that with these 3D congestion patterns a new approach to a notoriously difficult problem (predicting travel times in urban networks) is possible. Even though optimizing the clustering and the post-treatment operations is very important for larger networks with (much) more links and data, it should be noticed that (continuously) learning and updating the consensual patterns with new daily patterns are off-line steps that can be performed over the night (determining the 3D congestion maps for a new day) and over the weekend (updating the consensual patterns). The critical component for real-time travel time estimation is the matching between the current observations and the historical data included in the 3D consensual congestion maps. With our method, this operation is so fast that it can already be applied in much larger networks. In this paper, we do already hint at an important avenue to significantly cut computational costs for the original clustering operations. We constructed the 208 link graph of Amsterdam through *coarsening* the original 7512 link OSM network, using a constrained version of contraction hierarchy^44^ as explained in^20^. Network coarsening^45^ appears then as an efficient strategy to reduce the network size while preserving both network topology and the underlying data patterns. Also this strategy deserves further in-depth analysis and research.

Clearly, there are numerous other directions to further improve the methodologies behind the two contributions offered here. These relate for example to improve the underlying data processing methods, or to more advanced clustering techniques and matching procedures. Nonetheless, we believe the main results stand and touch upon a fundamental property of city traffic dynamics, and that is, that these dynamics may be more regular and predictable than expected. Consensual 3D speed maps enable us to extract the essence of large sets of detailed data to reveal the global picture about traffic dynamics in cities. We expect many applications of this concept not only for traffic monitoring and control but also for policy making and urban planning in general.

## Methods

### Initial dataset

In this study, link speed data are reconstructed from trip travel time observations. In Amsterdam, 127 cameras are recording license plates at the critical points of the major street networks (excluding freeways). This defines 314 single origin-destination (OD) pairs. For each OD pair the shortest path in distance is determined using the OpenStreetMaps GIS database^46^. The final network consists in all the links included in all the shortest paths, i.e. 7512 links in total. We apply an algorithm that merges together successive links in the same direction between two intersections. Internal links for intersections are also merged into a single node that only reproduces the available turning movements. At the end, the network has 208 links and 214 nodes^20^. The final step is to calculate the link speed information for 10 min time intervals from the individual travel times between OD pairs. We have a complete database of 35 days where we select the time period between 7am and 3 pm (morning peak hour and lunch time). The mean number of individual travel time records per day is 171000. Each individual travel time information provides both the departure and the arrival times. All travel time data that exceeds a given threshold added to the current moving average for a given OD are considered as outliers and then disregarded (7% in total). The remaining information are then matched to links assuming a constant travel speed. We used a 10 min time window for link speed data, meaning that all observations coming from vehicles that drive through a link during the same 10 min period are averaged into a single link speed value. A complete description of the data preparation can be found in^20^. Note that the data processing in this paper is not restricted to the data we used for the Amsterdam network but can be applied to any network with link speed information.

### Ncut algorithm

Ncut is a clustering algorithm based on a similarity matrix *S*(*i*, *j*) that defines the level of similarity between two elements *i* and *j* of the dataset^15^. In this paper, we use two different metrics to define the similarity: the Snake similarity^17^ when determining the original clustering for each day and the NMI, eq. 2, when gathering days with similar patterns. More details about the Snake similarity are provided in supplementary S1. The different steps of the Ncut algorithm are:

1. Calculate the diagonal matrix *D* of the similarity matrix *S*
2. Calculate the normalized Laplacian matrix $L={D}^{-\mathrm{1/2}}(D-S){D}^{-\mathrm{1/2}}$
3. Calculate the eigenvalues of *L* and increasingly order the eigenvectors with respect to the eigenvalues
4. To obtain a partition in 2^*m*^ clusters, select the 2nd to the ${\mathrm{(2}+m-\mathrm{1)}}^{th}$ eigenvectors in the ordered list. The splitting point here is equal to 0 meaning that we separate for each eigenvector the values >0 and ≤0. Each observation is then codified into a set of *m* binary values > or ≤0 depending on the related values in the eigenvectors. Each observation with the same codification falls into the same cluster.
5. When the targeted number of clusters is not a power of 2, take the closest higher value for 2^*m*^ that then apply a merge algorithm. Clusters with the closest similarities are iteratively merged two by two^17^.

### k-means and DBSCAN

Before running the k-means or the DBSCAN we first normalized each observation *i* defined by the following vector $({x}_{i},{y}_{i},{t}_{i},{v}_{i})$, where *x*_*i*_ and *y*_*i*_ are the geographical coordinates of the middle of a link, *t*_*i*_ defines the time period and *v*_*i*_ the speed value. Normalization is performed based on the global minimal and maximal values for all coordinates. Speed values are then overweighted by a factor 3 because this variable should play a predominant role during the clustering process. For both algorithms, the distance between two observations is assessed based on the Euclidean one. The details of k-means algorithm can be found in^21^. The only parameter is the number of targeted clusters. The DBSCAN (Density-based spatial clustering of applications with noise) has been proposed by Ester *et al*. in 1996^22^. It is a density-based clustering algorithm that groups together points that are close, i.e. within a circle of radius $\varepsilon$ (0.005 in our case). There is no targeted number of clusters but a minimal number of points to define a cluster (10 in our case). The algorithm stops when all points have been labeled. To obtain a given number of clusters, clusters are finally merged using the same algorithm as for the Ncut^17^. In practice both k-means and DBSCAN scripts have been retrieved from the MATLAB^©^ File Exchange website^47,48^.

### Data availability

All the data related to this study and its documentation are accessible using the following links: http://dittlab.tudelft.nl:8080/3DPartitioning or https://doi.org/10.6084/m9.figshare.5198566.

## Electronic supplementary material

Supplementary information

## References

[1] Brockmann D, Hufnagel L, Geisel T (2006). The scaling laws of human travel. Nature.

[2] GonzÃ¡lez M, Hidalgo C, Barabási A (2008). Understanding individual mobility patterns. Nature.

[3] Song C, Koren T, Wang P, Barabási A (2010). Modelling the scaling properties of human mobility. Nature Physics.

[4] Song C, Qu Z, Blumm N, Barabási A (2010). Limits of predictability in human mobility. Science.

[5] Zhao K, Musolesi M, Hui P, Rao W, Tarkoma S (2015). Explaining the power-law distribution of human mobility through transportation modality decomposition. Scientific reports.

[6] Ortuzar, J. & Willumsen, L. Modelling transport. *(**Wilsey,Chichester**)* (1994).

[7] Wilson A (1998). Land-use/transport interaction models:past and future. Journal of Transportation Economic Policy.

[8] Choukroun J (1975). A general framework for the development of gravity-type trip distribution models. Regional Science and Urban Economics.

[9] Lenormand M, Bassolas A, Ramasco JJ (2016). Systematic comparison of trip distribution laws and models. Journal of Transport Geography.

[10] Peng C, Jin X, Wong K, Shi M, Lio P (2012). Collective human mobility pattern from taxi trips in urban area. PLOS ONE.

[11] Wang P, Hunter T, Bayen A, Schechtner K, GonzÃ¡lez M (2012). Understanding road usage patterns in urban areas. Scientific reports.

[12] Anas A (1983). Discrete choice theory, information theory and the multinomial logit and gravity models. Transportation Research part B.

[13] Yang Y, Herrera C, Eagle N, González M (2014). Limits of predictability in commuting flows in the absence of data calibration. Scientific Reports.

[14] Alpaydin, E. *Ntroduction to Machine Learning–3rd edition* (MIT Press, 2014).

[15] Shi J, Malik J (2000). Normalized cuts and image segmentation. IEEE Transaction on Pattern Analysis and Machine Intelligence.

[16] Ji Y, Geroliminis N (2012). On the spatial partitioning of urban transportation networks. Transportation Research Part B.

[17] Saeedmanesh M, Geroliminis N (2016). Clustering of heterogeneous networks with directional flows based on “snake” similarities. Transportation Research Part B.

[18] Cover, T. M. & Thomas, J. A. *Elements of Information Theory**2nd Edition**(Wiley Series in Telecommunications and Signal Processing)* (Wiley-Interscience, 2006).

[19] Filkov V, Skiena S (2004). Integrating microarray data by consensus clustering. International Journal on Artificial Intelligence Tools.

[20] Lopez, C., Krishnakumari, P., Leclercq, L., Chiabaut, N. & van Lint, H. Spatio-temporal partitioning of the transportation network using travel time data. *Transportation Research Records* 14Â p. 10.3141/2623-11 (2017).

[21] MacQueen, J. B. Some methods for classification and analysis of multivariate observations. In Cam, L. M. L. & Neyman, J. (eds) *Proc. of the fifth Berkeley Symposium on Mathematical Statistics and Probability*, vol. 1, 281–297 (University of California Press, 1967).

[22] Ester, M., Kriegel, H.-P., Sander, J. & Xu, X. A density-based algorithm for discovering clusters in large spatial databases with noise. In *Proceedings of the Second International Conference on Knowledge Discovery and Data Mining*, KDD'96, 226–231 (AAAI Press, 1996).

[23] Yang F, Li T, Zhou Q, Xiao H (2017). Cluster ensemble selection with constraints. Neurocomputing.

[24] Ozay, M. Semi-supervised segmentation fusion of multi-spectral and aerial images. In 2014 *22nd International Conference on**Pattern Recognition*10.1109/icpr.2014.659 (IEEE 2014).

[25] Vlahogianni, E. I., Karlaftis, M. G. & Golias, J. C. Short-term traffic forecasting: Where we are and where we’re going. *Transportation Research Part C: Emerging Technologies* **43**, 3–19, 10.1016/j.trc.2014.01.005. Special Issue on Short-term Traffic Flow Forecasting (2014).

[26] Mori U, Mendiburu A, Álvarez M, Lozano JA (2015). A review of travel time estimation and forecasting for advanced traveller information systems. Transportmetrica A: Transport Science.

[27] Wang Y, Papageorgiou M, Messmer A (2006). Renaissance - a unified macroscopic model-based approach to real-time freeway network traffic surveillance. Transportation Research Part C: Emerging Technologies.

[28] Kumar BA, Vanajakshi L, Subramanian SC (2017). Bus travel time prediction using a time-space discretization approach. Transportation Research Part C: Emerging Technologies.

[29] Nantes A, Ngoduy D, Bhaskar A, Miska M, Chung E (2016). Real-time traffic state estimation in urban corridors from heterogeneous data. Transportation Research Part C: Emerging Technologies.

[30] Zhang X, Rice JA (2003). Short-term travel time prediction. Transportation Research Part C: Emerging Technologies.

[31] Rice J, Zwet Ev (2004). A simple and effective method for predicting travel times on freeways. IEEE Transactions on Intelligent Transportation Systems.

[32] Biswas, S., Chakraborty, S., Chandra, S. & Ghosh, I. Kriging-based approach for estimation of vehicular speed and passenger car units on an urban arterial. *Journal of Transportation Engineering* **143**10.1061/JTEPBS.0000031 (2017).

[33] Xu Y, Chen H, Kong Q-J, Zhai X, Liu Y (2016). Urban traffic flow prediction: A spatio-temporal variable selection-based approach. Journal of Advanced Transportation.

[34] Bahuleyan, H. & Vanajakshi, L. D. Arterial path-level travel-time estimation using machine-learning techniques. *Journal of Computing in Civil Engineering* **31** (2017).

[35] Huang W, Song G, Hong H, Xie K (2014). Deep architecture for traffic flow prediction: Deep belief networks with multitask learning. IEEE Transactions on Intelligent Transportation Systems.

[36] Wang J, Tsapakis I, Zhong C (2016). A space-time delay neural network model for travel time prediction. Engineering Applications of Artificial Intelligence.

[37] Van Lint JWC (2008). Online learning solutions for freeway travel time prediction. IEEE Transactions on Intelligent Transportation Systems.

[38] Lv Y, Duan Y, Kang W, Li Z, Wang FY (2015). Traffic flow prediction with big data: A deep learning approach. IEEE Transactions on Intelligent Transportation Systems.

[39] Yang, H., Dillon, T. S. & Chen, Y. P. Optimized structure of the traffic flow forecasting model with a deep learning approach. *IEEE Transactions on Neural Networks and Learning Systems* (2016). 10.1109/TNNLS.2016.2574840 27448371

[40] Bhaskar A, Tsubota T, Kieu LM, Chung E (2014). Urban traffic state estimation: Fusing point and zone based data. Transportation Research Part C: Emerging Technologies.

[41] Li L (2015). Robust causal dependence mining in big data network and its application to traffic flow predictions. Transportation Research Part C: Emerging Technologies.

[42] Laharotte, P.-A., Billot, R., El-Faouzi, N.-E. & Rakha, H. A. Network-wide traffic state prediction using bluetooth data. In *TRB 94th Annual Meeting Compendium of Papers, 15-3022* (Transportation Research Board, 2015).

[43] Fusco G, Colombaroni C, Isaenko N (2016). Short-term speed predictions exploiting big data on large urban road networks. Transportation Research Part C: Emerging Technologies.

[44] Geisberger, R., Sanders, P., Schultes, D. & Delling, D. Contraction hierarchies: Faster and simpler hierarchical routing in road networks. In McGeoch, C. C. (ed.) *Experimental Algorithms: 7th International Workshop, WEA 2008 Provincetown, MA, USA, May 30-June 1, 2008 Proceedings*, 319–333 (Springer, Berlin, Heidelberg, 2008).

[45] Chevalier, C. & Safro, I. Comparison of coarsening schemes for multilevel graph partitioning. In *Lecture Notes in Computer Science*, 191–205 (Springer Berlin Heidelberg, 2009).

[46] OpenStreetMap contributors. Planet dump retrieved from, https://planet.osm.org. https://www.openstreetmap.org (2017).

[47] Mo C. Kmeans algorithm retrieved on 2016-12-10 from, https://fr.mathworks.com/matlabcentral/fileexchange/24616-kmeans-clustering (2016).

[48] Yarpiz. DBSCAN Clustering Algorithm retrieved on 2016-12-10 from, https://fr.mathworks.com/matlabcentral/fileexchange/52905-dbscan-clustering-algorithm (2016).

