# Supplementary material for: Revealing the day-to-day regularity of urban congestion patterns with 3D speed maps
Source: Sci Rep. 2017 Oct 25;7:14029. doi: 10.1038/s41598-017-14237-8 (PMC5656590; doi:10.1038/s41598-017-14237-8)
Supplement: Supplementary file 1 — Supplementary information [file 41598_2017_14237_MOESM1_ESM.pdf]

Revealing the day-to-day regularity of urban congestion patterns with 3D speed maps

Clélia Lopez[1], Ludovic Leclercq[1,*], Panchamy Krishnakumari[2], Nicolas Chiabaut[1], Hans van Lint[2]

[1]Univ. Lyon, IFSTTAR, ENTPE, LICIT, Lyon, F-69675, France
[2]Delft University of Technology, CIGT, Delft, N-2600GA, The Netherlands
[*]corresponding author: ludovic.leclercq@ifsttar.fr

Supplementary information

S1 Calculating the Snake similarity matrix

The concept of *snakes* was first introduced by Saeedmanesh and Geroliminis (2016). A snake is an array of connected links. It is initialized by randomly selecting an initial link. New adjacent links are iteratively added to minimize at each step the link speed variance within the snake. The variance and the mean link speed of a snake at step N are denoted σ_N^2 *and* $\bar{x}_N$ and are defined as follows:

$$\sigma_N^2 = \frac{(N-1)\sigma_{N-1}^2 + (x_N - \bar{x}_N)(x_N - \bar{x}_{N-1})}{N}$$

$$\bar{x}_N = \frac{(N-1)\bar{x}_{N-1} + x_N}{N}$$

Where x_N is the speed value of the optimal link to add, i.e. the link among all adjacent links to the existing snake at step $N-1$ that is minimizing σ_N^2.

We use snakes to build the similarity matrix denoted S. The snake similarity s_{ij} between two links i and j is defined as follows:

$$s_{ij} = \sum_{k=1}^{N} p^k \; Card\left(W_{ik} \cap W_{jk}\right),$$

where $p^k \leq 1$ is the weight coefficient, W_i is the snake's sequence initialized by the link l_i, W_{ik} the sub-sequence of k first elements of W_i, and $Card(W_{ik} \cap W_{jk})$ is the number of common elements in two given sub-sequences. In our case of study, we set $p = 0.8$. Moreover, to reduce computational costs, snakes are truncated to 38% of the network size, i.e. when N is about 4000.

The normalized cut criterion (Shi and Malik, 2000) considers (i) the total inter-cluster dissimilarity and (ii) the intra-cluster similarity. Ncut was initially proposed for image segmentation. In that case, Shi and Malik (2000) set the similarity s_{ij} between two nodes (pixels) i and j to 0 when the number of intermediate nodes connecting *i* and *j* is higher than r. Note that in our application nodes are the transportation network links and edges are connecting adjacent network links. Shi and Malik (2000) show that the calibration of $r > 0$ returns more than a single-connected component for each cluster. To obtain a single-connected component in each cluster we should then set $r = 0$. However, if this condition

appears to be sufficient when studying fully connected graphs (like in image segmentation where pixels are connected to all its neighbors), it is no longer the case when studying transportation networks. When setting $s_{ij} = 0$ when two network links that are not adjacent in a transportation network, we observe that the clusters are more identified as communities, i.e. groups of links having high edges density intra-cluster and low edge density inter-cluster, than as homogeneous regions. To tackle such a problem, Saeedmanesh and Geroliminis (2016) propose to use a complete similarity matrix using the snake similarity for every pair of links. Thus, the Ncut algorithm based on snake similarity (S-Ncut) does not guarantee full intra-cluster connectivity of nodes. S-Ncut requires a post-processing algorithm just like k-means and DBSCAN. The description of the algorithm is provided in section (S3).

S2 Rationales behind the choice of the α value – weighting of speed vs. space and time values

To perform network clustering based on Euclidian distances (DBSCAN and k-means methods), after normalizing each factor (speed, space-x, space-y and time), we weight speed three times more heavily (α=3) compared to all other three variables. The weighting factor is unique for all situations. We describe here the rationales for this choice in details and provide comparisons with other α values. For clarifications about post-treatment operations, readers are referred to the following section S3.

First, it should be noted that α only influences the results from the DBSCAN and k-means methods as Ncut is not using Euclidean distances but the snake similarity matrix. The results are sensitive to α for the following reasons. When α is low (close to 1), the clustering favors closeness between links, i.e. the spatial distance. The time-distance is not of great influence here as the points are evenly distributed in time for all links. So, the resulting clusters will not be very homogeneous in speeds: TV_n values are high and CCD_n values are low, see figures S2(a) and S2(c). When α is very high, the speed values dominate and the clustering results are very good in terms of intra-cluster homogeneity (low TV_n values) and inter-cluster dissimilarity (high CCD_n values). However, clusters can gather links from mostly anywhere in the network as only speed values matter. If we assume that close links in distance are more likely to be connected than distant ones, the immediate corollary is that post-treatment will be much easier and quicker in terms of computational times when α is low than when it is high. Furthermore, the discrepancies between the clustering results before and after the post-treatment quickly increase with α. This is confirmed by comparing figures S2(a) and S2(b) for TV_n values and figures S2(c) and S2(d) for CCD_n values. Figures S2(e) and S2(f) show directly the values of the mean variations of TV_n and CCD_n values after post-treatment for all 35 days. A very important observation is that the TV_n and CCD_n values, see figures S2(b) and S2(d), stabilize after post-treatment when α is higher than 6, as the post-treatment annihilates any gains coming from increasing α during the initial clustering operations. The same figures show that α values lower or equal to 2 fail in the end (after post-processing) to provide good clustering results as the intra-cluster homogeneity is poor (high TV_n values) and so is the inter-cluster dissimilarity (low CCD_n values).

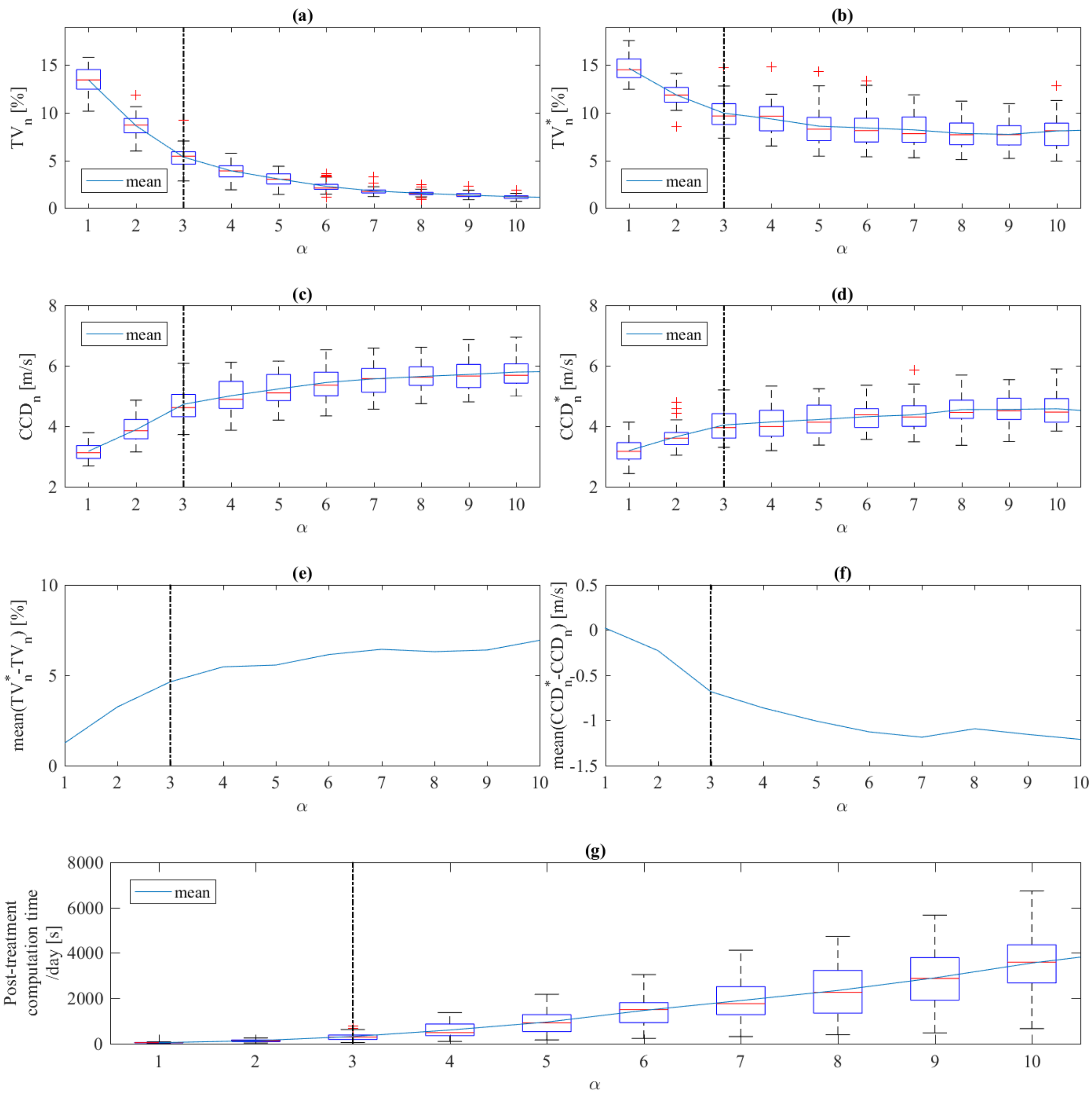

Figure S2 – (a-b) Total variance (TV_n) with respect to the α values for all 35 days and 9 clusters before (a) and after (b) post-treatment; (c-d) Connected cluster dissimilarity (CCD_n) with respect to the α values for all 35 days and 9 clusters before (c) and after (d) post-treatment; (e-f) Mean variations of TV_n (e) and CCD_n (f) values with respect to α values for all 35 days and 9 clusters before and after post-treatment. TV^*_n and CCD^*_n are the values after post-treatment while TV_n and CCD_n are the values before post-treatment; (g) Computation times for post-treatment operations with respect to the α values for all 35 days and 9 clusters.

So, the results of this sensitivity analysis show that α should be between 3 and 6 for the best. We can further elaborate on the choice of α=3 in the paper. First, Figure S2(g) shows a quick increase of the computation times when α is increasing because the post-treatment algorithm takes more time to re-organize the clusters to ensure full intra-cluster connectivity. This is again because when α is increasing the initial clustering focuses more and more on speed values only and gathers together links that are quite far (and that are thus more likely not to be connected). For example, the mean computational time for one day and the post-treatment is equal to 320 s, 604 s, 1080 s and 1800 s when α increases from 3 to 6. As we had to repeat the clustering operations a huge number of times in the paper to test different factors like the influence of the clustering method, the number of clusters and the day, a value

of α=3 permitted us to speed up the calculations significantly. Second, for the same reason, when α is increasing the final results are more dependent on the post-treatment algorithms than on the initial clustering. Since clustering algorithms have been extensively studied in the literature, whereas the post-treatment algorithm has been self-designed here, choosing α=3 permitted us to put more weight on the results of the initial clustering while having final TV_n and CCD_n values (after post-treatment) that are close to what is obtained with higher α, see Figures S2(b) and S2(d). Third, it is important to recall here that the results of k-means method with α=3 clearly outperforms the S-Ncut for all 35 days, see Figure 2b in the paper. So, α=3 looks sufficient to have good clustering results while balancing the computational times. In follow up work it would be interesting to see if the results of the travel times estimation based on the consensual clustering shapes could be further improved by taking α between 4 and 6. We nonetheless would like to stress here that the results with α=3 are already very good and certainly impressive when considering the simplicity of the method and the spatial coverage that we can achieve (basically the whole city area).

S3 Post-treatment of clustering results to ensure connectivity in each cluster

The three-step post-treatment algorithm (Lopez et al., 2017) is described as follows:

Step 1 – Initialization. The connected components (CCs) are identified within each cluster. A CC is a sub-graph where links are connected to each other. For each cluster, corresponding CCs are sorted in decreasing order based on the number of links.

Step 2 – Allocation. The biggest CC in each cluster is assigned as the initial cluster. The remaining CCs from every cluster are considered as candidates for merging.

Step 3 – Merging. Assign iteratively the candidate CC to an existing cluster in order to minimize the increase of the link speed variance within this cluster. The constraint is the connectivity between a given candidate CC and the candidate cluster. Note that the size of the initial clusters grow as CCs are merged iteratively. The merging process to reach the desired number of clusters has been initially proposed by Ji and Geroliminis (2012).

Figures S3(a) and S3(b) show respectively the effect of post-treatment on TV_n and CCD_n for a particular day and different numbers of clusters. This effect is assessed based on the variation of the TV_n and CCD_n after and before the post-treatment. It appears that he post-treatment has little effect on TV_n and CCD_n results when S-Ncut is applied. For k-means and DBSCAN, the post-treatment can either deteriorate or improve the TV_n and CCD_n. Note that a deterioration means a positive value for $TV_n^* - TV_n$ and a negative value for $CCD_n^* - CCD_n$, where the indexes with a star (*) correspond to the values after post-treatment. A deterioration is mostly observed from k-means results whatever the number of clusters is. DBSCAN results look to be often improved by the post-treatment in particular when the number of clusters is large (higher than 10). However, it is important to consider not only the variations of the indicators but also the final values. Furthermore, no definitive conclusion can be given from a single day.

Figures S3(c) and S3(d) show a more comprehensive look at the distribution of the variations of TV_n and CCD_n after and before post-treatment for all 35 days for number of cluster equal to 9 (optimal number). This confirms that S-Ncut results are not significantly modified by the post-treatment as the variations of TV_n values are close to 0% for most days. The post-treatment increases TV_n values in mean by 0.9% for DBSCAN and by 4% for k-means, see

Figure S3(c). This is not surprising as adding requirement (i) about full connectivity between links within each cluster require to reshape the clusters and deteriorate the optimal allocation that was made by the clustering algorithm without considering this constraint. S-Ncut results lead to very small discrepancies compared to the other two methods because S-Ncut resorts to a similarity matrix that already partly accounts for connectivity between links, while DBSCAN and k-means only account for proximity through the distance between links. Similar trends are observed for the CCD_n values but to a lesser extent. Again, the variations with S-Ncut are mostly close to 0 for all days, see figure S3(d). The variations for DBSCAN are close to 0 in mean but the amplitude is important. The variations for k-means are negative and equal in mean to -0.6 m/s, see figure S3(d). Thus, the main conclusion here is that the post-treatment has no impact for S-Ncut and mainly deteriorates TV_n values for k-means and DBSCAN. The variations are much pronounced for k-means but remain below 5% for most days (this is one of the reason for our choice for α=3 when weighting the speed values, see supplementary S2). However, DBSCAN and k-means results in one hand and N-Cut results in the other hand cannot be directly compared before post-treatment as N-Cut already favors connectivity between links through the definition of the similarity matrix. Furthermore, the DBSCAN method seems to less deteriorate TV_n and CCD_n values but the results from the initial clustering are much better with the k-means.

So, because requirement (i) should be fulfilled in the end by all the methods what matters is the comparison of the TV_n and CCD_n values after post-treatment. There, the k-means outperforms the other two methods, see Figure 2b in the paper.

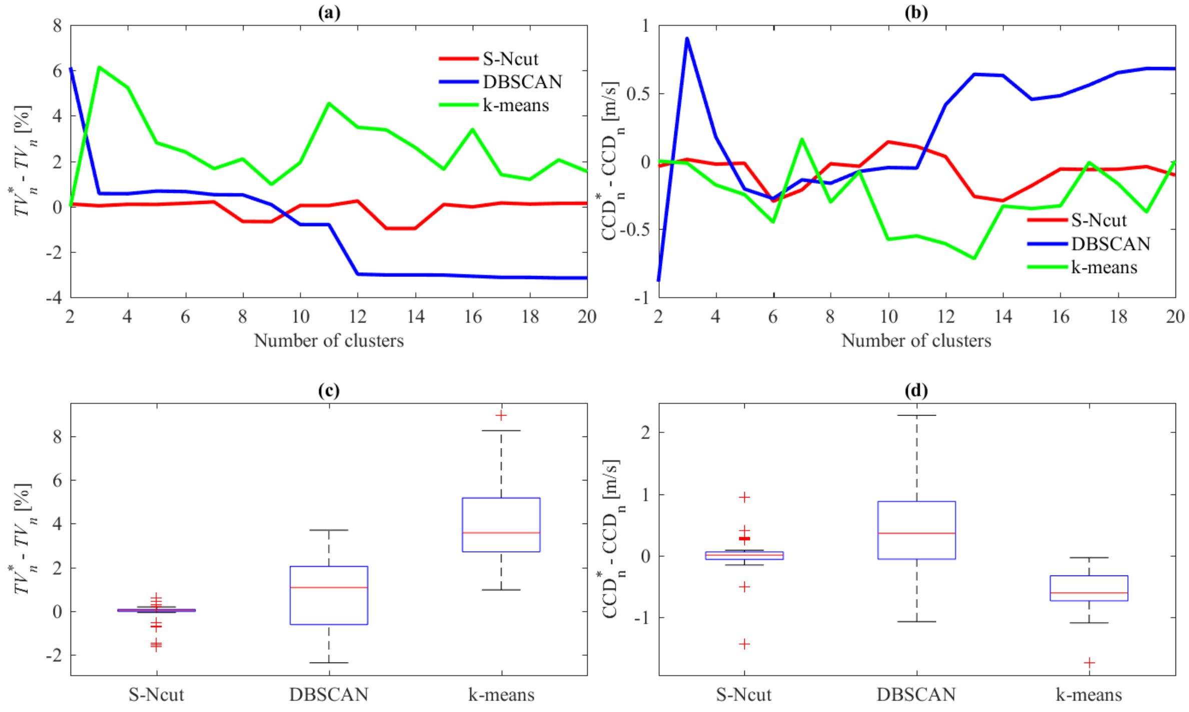

Figure S3 – (a-b) Variations of total variance (TV_n) and the connected cluster dissimilarity (CCD_n) after the post-treatment with respect to the number of clusters for different methods and a particular day. TV_n^* and CCD_n^* are the values after post-treatment while TV_n and CCD_n are the values before post-treatment; (c-d) Distributions of ($TV_n^* - TV_n$) and ($CCD_n^* - CCD_n$) for all 35 days, a number of cluster equal to 9 and the different clustering methods.

S4 Defining a common network for all days

After mapping the shortest path to the Amsterdam Geographic Information System (GIS) network, there are still 7512 links in the link network, which is quite large. Hence, we employ network coarsening techniques to remove nodes that satisfy certain criteria (Lopez et al., 2017). In this work, the nodes are removed based on the link weights. If two links have the same weight, the node connecting them is removed. The weight for each link, in our case, is the estimated speed of each link. As we only require speed for one time slice for assigning the weights for coarsening, a peak period time slice (4pm) was chosen. This is because the network will exhibit most variance during the peak period during which the largest differences in speed link speed occur. If we chose a non-peak period, these variances will be smoothed out. As for each day, the network state (speed of each link in the network) at 4pm is different, we will have 35 different coarsened networks for the 35 days. This is, especially, predominant in the case of weekends where some links of the network have too few observations compared to the other days. Thus, we need to extract a common network from these 35 coarsened networks. The common network is the intersection of the links in the 35 coarsened networks so that the shared dynamics of the data that is present in every day is captured. Meta-clustering methods require the same dataset which is why a common network is necessary.

S5 Quality of the classification of all days

This supplementary section (S5) presents more details about the quality of the classification of days intro groups. As explained in the paper, we use the NMI index to assess the similarity between two original clustering shapes related to two different days i and j. Let recall that the results of the initial clustering of day i can be put into a single ordered vector of all observations π_i, whose values are the cluster ID. Figure S5(a) presents the distribution NMI values for all couples of days among the total population, i.e. the 35 days. The distribution looks Gaussian with a mean and a standard deviation respectively equal to 0.59 and 0.056. The extreme values are 0.43 and 0.78.

Figure S5(b) shows now for each training set (12 replications) the quality of the clustering into two or four groups based on Ncut. The quality is calculated as follows:

$$quality = \frac{1}{N_p}\sum_{k=1}^{N_p}\frac{1}{C_{N_k}^2}\sum_{i=1}^{N_k}\sum_{\substack{j=i+1\\ i\neq j}}^{N_k} NMI(\pi_i,\pi_j)$$

where N_p is the number of groups, N_k is the number of days in group k. This indicator basically represents the global average of the mean NMI for each group. The higher it is, the more homogeneous all groups are. Figure S5(b) demonstrates that considering 4 groups significantly improves the quality of the day clustering compared to only two groups for 11 training sets over 12 (the exception is replication No. 2). This is why we are using 4 groups in the paper. Note that the mean NMI value for all days and each training set is also presented in figure S5(b) (1 group case) to show that the quality of the partitioning really improves when using 4 groups.

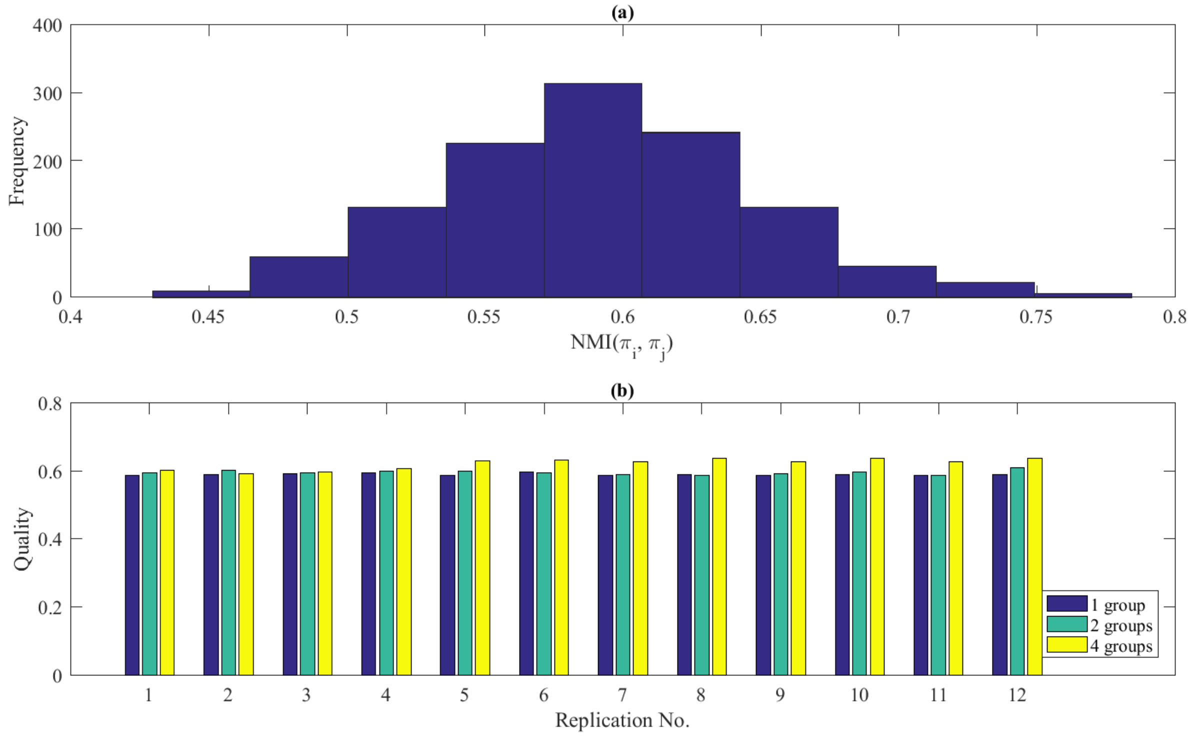

Figure S5 – (a) Similarity distribution between all days and (b) quality measurement all training sets

Finally, table S5 permits to better assess the quality of the partitioning into 4 groups for all training sets. It presents, for all replications, the mean and the amplitude, i.e. the difference between the max and the min, of the NMI values related to all day couples within each group (*G1* to *G4*). These values have to be compared with the same index but for all the days without partitioning (*All*). First, it appears that the amplitudes within groups are significantly lower than the amplitude of the total population of days for all replications. The mean amplitude for all groups and all replications is 0.19, which is nearly half of the mean amplitude for the total population. Second, most of mean NMI values are higher than 0.59 (90% in total). Recall that 0.59 is the median of the original distribution of the NMI values as shown in figure S5(a), meaning that 50% of the NMI values over the global population of days are below 0.58. Combined with low amplitudes within groups, this confirms that the partitioning into groups lead to close NMI values for days belonging to the same group, meaning similar clustering shapes. So, the analysis of table S5 permits to conclude that the 4 groups obtained after the classification of the 28 days are homogeneous as expected for all training sets.

Replications	mean NMI value within groups					Amplitude (Max-Min) of NMI values within groups				
	All	G1	G2	G3	G4	All	G1	G2	G3	G4
1	0.5879	0.6052	0.5769	0.6550	0.5689	0.3548	0.1399	0.2807	0.2088	0.1355
2	0.5898	0.5772	0.5838	0.6012	0.6038	0.3093	0.2158	0.1674	0.2430	0.2226
3	0.5922	0.6223	0.6114	0.5833	0.5736	0.3288	0.2679	0.2203	0.0988	0.2747
4	0.5934	0.6078	0.6590	0.5661	0.5994	0.3548	0.1838	0.1820	0.2807	0.1820
5	0.5870	0.6052	0.6123	0.6550	0.6498	0.3548	0.2313	0.2174	0.0923	0.2493
6	0.5960	0.6218	0.6253	0.6358	0.6438	0.3239	0.1309	0.2318	0.0961	0.2143
7	0.5879	0.6192	0.6079	0.6540	0.6308	0.3548	0.1643	0.2490	0.2056	0.0869
8	0.5898	0.6524	0.6080	0.6363	0.6463	0.3093	0.1891	0.2543	0.1918	0.1365
9	0.5879	0.6192	0.6079	0.6540	0.6308	0.3548	0.1643	0.2490	0.2056	0.0869
10	0.5898	0.6524	0.6080	0.6363	0.6463	0.3093	0.1891	0.2543	0.1918	0.1365
11	0.5879	0.6192	0.6079	0.6540	0.6308	0.3548	0.1643	0.2490	0.2056	0.0869
12	0.5898	0.6524	0.6080	0.6363	0.6463	0.3093	0.1891	0.2543	0.1918	0.1365

Table S5 – The mean and the amplitude of NMI values per group compared to the total population of days (All) for all training sets (replications)

S6 Travel time estimation on the training and validation set: absolute errors

This supplementary section (S6) presents the travel time prediction errors with absolute values. Figure S6(a) is equivalent to figure 4(b) in the paper and figure S6(b) is equivalent to figure 4(e). For the training set, the mean and median values of the errors are respectively equal to -0.6 and -0.3 min for method M1. The values are respectively -0.7 and -0.4 min for both M2 and M3. With respect to the distribution the 25th and the 75th percentiles are -2.1 min and 1 min (M1), -2.3 min and 1 min (M2) and -2.4 min and 1.1 min (M3). The 10th and 90th percentiles are -4 min and 2.3 min (M1), -4 min and 2.4 min (M2) and -4.3 min and 2.5 min (M3). All these confirm the conclusions given in the paper, which is that (i) most of the errors come from averaging speed inside a cluster and (ii) the consensus congestion map for a group (consensus cluster shape and mean speed of all days within each cluster) is sufficient to provide accurate travel time predictions (comparison of M3 vs. M1).

In figure S6(b), the mean and the median values are -0.7 min and -0.3 min, the 25th and 75th percentiles are -2.4 min and 1.2 min and the 10th and the 90th percentiles are -4.3 min and 2.5 min. Again, the conclusions of the paper are confirmed here: despite its simplicity and its very low computational cost, the travel time prediction method that consists in assigning new observations to an historical consensual congestion map lead to accurate travel time predictions for most trips.

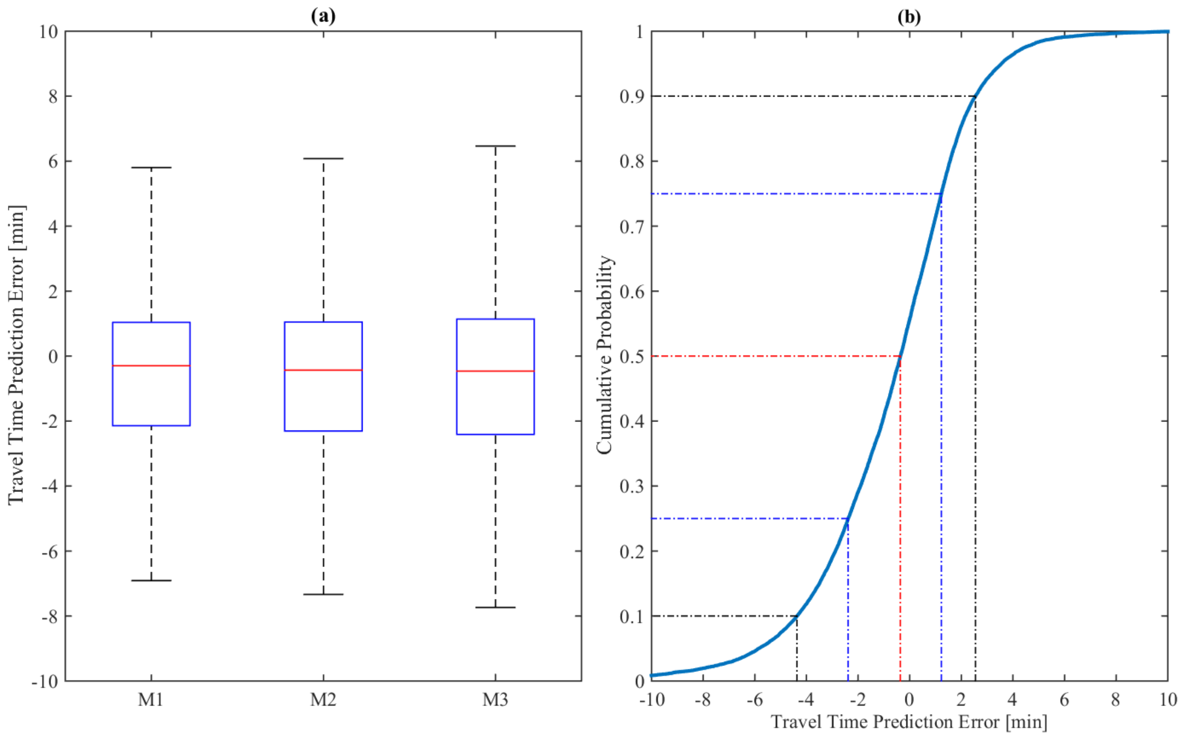

Figure S6 – (a) Travel time estimation errors for all probe trips and all training days considering the three estimation methods. (b) Distribution of the travel time estimation errors for the 10 probe trips, all validation days and all departure times.

References

Ji, Y., Geroliminis, N., 2012. On the spatial partitioning of urban transportation networks. *Transportation Research Part B*, 46:1639-1656.

Saeedmanesh, M., Geroliminis, N., 2016. Clustering of heterogeneous networks with directional flows based on “Snake” similarities. *Transportation Research part B*, 91:250-269.

Shi, J., Malik, J., 2000. Normalized cuts and image segmentation. *IEEE Transactions on Pattern Analysis and Machine Intelligence*, 22(8):888-905.

Lopez, C., Krishnakumari, P., Leclercq, L., Chiabaut, N. & van Lint, H. Spatio-temporal partitioning of the transportation network using travel time data. *Transportation Research Record*. 14 p. (2017). DOI http://dx.doi.org/10.3141/2623-11
